# Cytogeography of the *Humifusa* clade of *Opuntia* s.s. Mill. 1754 (Cactaceae, Opuntioideae, Opuntieae): correlations with pleistocene refugia and morphological traits in a polyploid complex

**DOI:** 10.3897/CompCytogen.v6i1.2523

**Published:** 2012-02-14

**Authors:** Lucas C. Majure, Walter S. Judd, Pamela S. Soltis, Douglas E. Soltis

**Affiliations:** 1 Department of Biology, University of Florida, Gainesville, FL, 32611, U.S.A.; 2 Florida Museum of Natural History, University of Florida, Gainesville, FL, 32611-7800, U.S.A.

**Keywords:** Cactaceae, chromosome numbers, *Opuntia humifusa*, *Opuntia macrorhiza*, Pleistocene refugia, polyploid complex, polyploidy

## Abstract

Ploidy has been well studied and used extensively in the genus *Opuntia* to determine species boundaries, detect evidence of hybridization, and infer evolutionary patterns. We carried out chromosome counts for all members of the *Humifusa* clade to ascertain whether geographic patterns are associated with differences in ploidy. We then related chromosomal data to observed morphological variability, polyploid formation, and consequently the evolutionary history of the clade. We counted chromosomes of 277 individuals from throughout the ranges of taxa included within the *Humifusa* clade, with emphasis placed on the widely distributed species, *Opuntia humifusa* (Raf.) Raf., 1820 s.l. and *Opuntia macrorhiza* Engelm., 1850 s.l. We also compiled previous counts made for species in the clade along with our new counts to plot geographic distributions of the polyploid and diploid taxa. A phylogeny using nuclear ribosomal ITS sequence data was reconstructed to determine whether ploidal variation is consistent with cladogenesis. We discovered that diploids of the *Humifusa* clade are restricted to the southeastern United States (U.S.), eastern Texas, and southeastern New Mexico. Polyploid members of the clade, however, are much more widely distributed, occurring as far north as the upper midwestern U.S. (e.g., Michigan, Minnesota, Wisconsin). Morphological differentiation, although sometimes cryptic, is commonly observed among diploid and polyploid cytotypes, and such morphological distinctions may be useful in diagnosing possible cryptic species. Certain polyploid populations of *Opuntia humifusa* s.l. and *Opuntia macrorhiza* s.l., however, exhibit introgressive morphological characters, complicating species delineations. Phylogenetically, the *Humifusa* clade forms two subclades that are distributed, respectively, in the southeastern U.S. (including all southeastern U.S. diploids, polyploid *Opuntia abjecta* Small, 1923, and polyploid *Opuntia pusilla* (Haw.) Haw., 1812) and the southwestern U.S. (including all southwestern U.S. diploids and polyploids). In addition, tetraploid *Opuntia humifusa* s.l., which occurs primarily in the eastern U.S., is resolved in the southwestern diploid clade instead of with the southeastern diploid clade that includes diploid *Opuntia humifusa* s.l. Our results not only provide evidence for the polyphyletic nature of *Opuntia humifusa* and *Opuntia macrorhiza*, suggesting that each of these represents more than one species, but also demonstrate the high frequency of polyploidy in the *Humifusa* clade and the major role that genome duplication has played in the diversification of this lineage of *Opuntia* s.s. Our data also suggest that the southeastern and southwestern U.S. may represent glacial refugia for diploid members of this clade and that the clade as a whole should be considered a mature polyploid species complex. Widespread polyploids are likely derivatives of secondary contact among southeastern and southwestern diploid taxa as a result of the expansion and contraction of suitable habitat during the Pleistocene following glacial and interglacial events.

## Introduction

Ploidy has a long tradition of utility for illuminating species boundaries, hybrid zones, and interspecific relationships among plants (e.g., Stace 2000). Knowing the ploidal levels of taxa used in phylogenetic analyses can also aid in detecting potential hybridization events through incongruence in reconstructions using biparentally inherited nuclear loci (Ionta et al. 2007, Soltis et al. 2008). Researchers have frequently used cytological data to help understand species evolution and delimitations in the nopales or prickly pear cacti, i.e., the genus *Opuntia* (Pinkava and McLeod 1971, Pinkava et al. 1973, 1977, 1985, Weedin and Powell 1978, Pinkava and Parfitt 1982, Weedin et al. 1989, Pinkava et al. 1992, Powell and Weedin 2001, 2004). Subfamily Opuntioideae (*Opuntia* s.l., as previously recognized; Benson 1982) is known to have the highest number of polyploids in Cactaceae (Cota and Philbrick 1994, Pinkava 2002), and *Opuntia* s.s. is well known for interspecific hybridization (e.g., Grant and Grant 1982, Griffith 2003) and subsequent genome duplication (Pinkava 2002, L.C. Majure (LCM), R. Puente (RP), P. Griffith (PG), W.S. Judd (WSJ), P.S. Soltis (PSS), D.E. Soltis (DES) unpubl. data).

The significance of polyploidy in plant evolution and speciation has long been recognized (Stebbins 1940, 1950, 1971; Swanson 1957, DeWet 1971, Harlan and DeWet 1975, Grant 1981, Leitch and Bennett 1997, Ramsey and Schemske 1998, Adams and Wendel 2005, Tate et al. 2005, Doyle et al. 2008, Soltis and Soltis 2009, Jiao et al. 2011). As stated by Stebbins (1950), p. 369), “polyploidy … is one of the most rapid methods known of producing radically different, but nevertheless vigorous and well-adapted genotypes.” Polyploidy also is considered one of the unequivocal means of true sympatric speciation (Futuyma 1998, Otto and Whitton 2000) and is considered to be common in plants (Stebbins 1940, DeWet 1971, Ramsey and Schemske 1998, Tate et al. 2005). For example, virtually all major clades of angiosperms have undergone one or more episodes of genome duplication (Soltis and Soltis 2009). Likewise, polyploidy is very important throughout Cactaceae (Pinkava 2002), and within *Opuntia* s.s., polyploids previously have been recorded in *Opuntia humifusa* (Raf.) Raf., 1820, and relatives (Bowden 1945a, b, Pinkava et al. 1985, Powell and Weedin 2004, Baker et al. 2009a, b, Majure and Ribbens in press) of the *Humifusa* clade (sensu LCM, RP, PG, WSJ, PSS, DES unpubl. data).

There are currently six species recognized in the *Humifusa* clade, *Opuntia abjecta* Small, 1923, *Opuntia humifusa*, *Opuntia macrorhiza* Engelm., 1850, *Opuntia pottsii* Salm-Dyck, 1849, *Opuntia pusilla* (Haw.) Haw., 1812, and *Opuntia tortispina* Engelm. & J.M. Bigelow, 1856 (Pinkava, 2003; LCM unpubl. data). The *Humifusa* clade is distributed widely from the western U.S. and northern Mexico (represented by *Opuntia macrorhiza* s.l., *Opuntia pottsii*, and *Opuntia tortispina*) and throughout the eastern U.S. including the upper Midwest (e.g., Michigan, Minnesota, Wisconsin) and southern Ontario (Benson, 1982; represented by *Opuntia abjecta*, *Opuntia humifusa* s.l., *Opuntia macrorhiza* s.l., and *Opuntia pusilla*).

*Opuntia humifusa* s.l. is composed of numerous morphological entities that have been recognized in certain taxonomic treatments as different species (see Small 1933). Throughout its range, *Opuntia humifusa* s.l. has been divided into as many as 14 taxa (Britton and Rose 1920, Small 1933, Benson 1982, Majure and Ervin 2008). Thus, *Opuntia humifusa* s.l. is occasionally referred to as a species complex (Doyle 1990). Currently, two taxa are recognized in *Opuntia humifusa* s.l. (*Opuntia humifusa* var. *ammophila* (Small) L.D. Benson and *Opuntia humifusa* var. *humifusa*; Pinkava 2003). Likewise, *Opuntia macrorhiza* has been divided into as many as 11 taxa (see Benson 1982). *Opuntia macrorhiza* was previously considered a variety of *Opuntia humifusa* (see Benson 1962; see Table 1 for synonyms of *Opuntia humifusa* s.l. and *Opuntia macrorhiza* s.l. sampled in this study), *Opuntia pottsii* was considered a variety of *Opuntia macrorhiza*, and *Opuntia tortispina* was placed in synonymy with *Opuntia macrorhiza* (Benson 1982).

**Figure 1.**
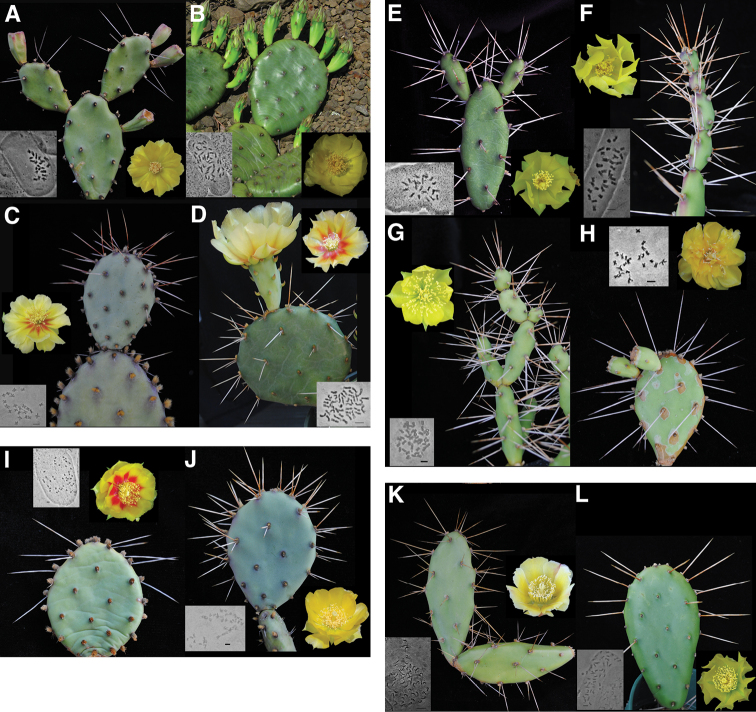
Selected taxa in the *Humifusa* clade with associated chromosome squashes **A** diploid *Opuntia humifusa* (*Opuntia lata*) *LCM 4106* **B** tetraploid *Opuntia humifusa* s.s. *LCM 3810* **C** diploid *Opuntia macrorhiza* (*Opuntia xanthoglochia*) *LCM 1983* **D** tetraploid *Opuntia macrorhiza* *LCM 3510* **E** diploid *Opuntia pusilla* *LCM 753* **F** triploid *Opuntia pusilla* *LCM 1033* **G** tetraploid *Opuntia pusilla* *LCM 3700* **H** diploid *Opuntia abjecta* *LCM 3908* **I** tetraploid *Opuntia humifusa* (*Opuntia cespitosa*) *LCM 2610* **J** tetraploid *Opuntia humifusa* (*Opuntia nemoralis*) *LCM 4204* **K** pentaploid *Opuntia ochrocentra* *LCM 3907* and **L** tetraploid *Opuntia humifusa* (*Opuntia pollardii*) *LCM 769*. Bars on photomicrographs = 5μm.

*Opuntia pusilla* has been divided into several species: *Opuntia drummondii* Graham, 1841, *Opuntia frustulenta* Gibbes, 1858, *Opuntia impedita* Small, 1923, *Opuntia pes-corvi* LeConte, 1857, and *Opuntia tracyi* Britton, 1911 (Britton and Rose 1920, Small 1933); however, Benson (1982) placed them in synonymy under the name *Opuntia pusilla*. *Opuntia triacantha* (Willd.) Sweet, 1826, also has been divided into several species, i.e., *Opuntia abjecta* of the Florida Keys, *Opuntia militaris* Britton & Rose, 1919, of Cuba, and *Opuntia triacantha* from different parts of the Greater and Lesser Antilles (Britton and Rose 1920), but all of these have since been placed in synonymy within *Opuntia triacantha* (Benson 1982). Phylogenetic and morphological studies have indicated that *Opuntia abjecta* is not even in the same clade as *Opuntia triacantha* (LCM, WSJ unpubl. data) and so here is treated as *Opuntia abjecta*.

Contributing to the confusing taxonomic history of this clade is the high degree of morphological variation exhibited by most taxa, the lack of complete sampling throughout the range of the clade, the absence of cytological and phylogenetic evidence, reliance on poorly prepared and sparse herbarium collections (Majure and Ervin 2008, LCM unpubl. data), and hybridization and polyploidy (Benson 1982, Rebman and Pinkava 2001). Careful examination of morphological characters across the geographic range of the widely distributed *Opuntia humifusa* s.l. and *Opuntia macrorhiza* s.l. reinforces the hypothesis that hybridization may have preceded the origin of geographical morphotypes, because morphological characters displayed by certain taxa appear to be introgessive between *Opuntia humifusa* s.l. and *Opuntia macrorhiza* s.l. (Table 2). For instance, *Opuntia cespitosa* Raf., 1830, from the eastern U.S. and recently recognized by Majure and Ervin (2008), has yellow tepals that are basally tinged crimson- to orange-red, a characteristic typical of *Opuntia macrorhiza* and occasionally *Opuntia tortispina* from western North America (Benson 1982, Pinkava 2003, Powell and Weedin 2004), but the spine characters of *Opuntia cespitosa* are typical of *Opuntia humifusa* s.l. (see Majure and Ervin 2008).

Although chromosome counts have been reported for many of the *Opuntia* taxa from the southwestern U.S. and other areas (Stockwell 1935, Spencer 1955, Pinkava and McLeod 1971, Pinkava et al. 1973, 1977; Weedin and Powell 1978, Pinkava and Parfitt 1982, Pinkava et al. 1985, Weedin et al. 1989, Pinkava et al. 1992, Powell and Weedin 2001, Pinkava 2002, Negrón-Ortiz 2007, Segura et al. 2007, Baker et al. 2009a, b), few chromosome counts have been reported for taxa of *Opuntia* in the eastern and midwestern U.S. (Majure and Ribbens in press), and most of those taxa belong to the *Humifusa* clade. Bowden (1945a, b), Hanks and Fairbrothers (1969), Doyle (1990), and Baker et al. (2009 a, b) have all made counts of members of the *Humifusa* clade from the eastern U.S. Bowden (1945a, b), Doyle (1990), and Baker et al. (2009a) recorded diploid (2*n* = 22) and tetraploid (2*n* = 44) material of *Opuntia humifusa* from the eastern U.S., and Bowden (1945a) recorded tetraploid (2*n* = 44) material of *Opuntia impedita* (currently syn. of *Opuntia pusilla*). Hanks and Fairbrothers (1969) recorded an aneuploid number for *Opuntia humifusa* (2*n* = 17, 19) likely in error, since aneuploids are very rare in Cactaceae (Pinkava 2002). Majure and Ribbens (in press) recorded tetraploids of *Opuntia humifusa* s.l. and *Opuntia macrorhiza* s.l. from the Midwest, suggesting that the northernmost populations of those taxa are polyploid. *Opuntia macrorhiza*, *Opuntia pottsii*, and *Opuntia tortispina* have all been counted extensively in the southwestern U.S. (Pinkava and McLeod 1971, Pinkava et al. 1973, Pinkava et al. 1977, Pinkava et al. 1992, Pinkava et al. 1998, Powell and Weedin 2001, Powell and Weedin 2004), where *Opuntia macrorhiza* and *Opuntia pottsii* have been recorded exclusively as tetraploids, and *Opuntia tortispina* has been recorded as either tetra- or hexaploid.

Chromosome counts reported for species in the *Humifusa* clade do not encompass all of the taxa within the range of the clade nor the wide distributions exhibited by several of the more common species. To further our understanding of species complexes and the evolution of polyploids within those complexes, cytological data are needed from the entire distribution of a given species (Babcock and Stebbins 1938, Stebbins 1942, Stebbins 1950). Thus, an in-depth study of the distribution of cytotypes and correlations between cytotypes and morphology is desperately needed in order to aid in the delimitation of potentially unrecognized and cryptic species and to elucidate relationships in the *Humifusa* clade.

Here we present chromosome counts for all taxa considered to be part of the *Opuntia humifusa* complex and all taxa of the *Humifusa* clade (LCM, WSJ, PSS, DES, unpubl. data) and provide counts throughout most of the known ranges of all taxa to determine the geographic structure of ploidy and differences in ploidy among morphologically distinct taxa. We also reconstruct a phylogeny of diploid and polyploid members of the *Humifusa* clade based on nrITS data to investigate the relationship between geographic distribution and evolutionary relationships. We provide counts for another common species in the southeastern U.S., *Opuntia stricta* (Haw.) Haw., 1812, because it has been hypothesized to hybridize with members of the *Humifusa* clade (Benson 1982). In addition, ploidy of the putative hybrid between *Opuntia abjecta* and *Opuntia stricta*, i.e., *Opuntia ochrocentra* Small, 1923, was analyzed. Ploidy determinations of the *Humifusa* clade, coupled with morphological character analysis and further molecular phylogenetics, will aid in the delimitation of species in the group and in determining the origin and evolutionary significance of polyploidy in this clade.

## Material and methods

*Chromosome counts – *Methods follow those of Majure and Ribbens (in press). Briefly, root tips were collected from early morning throughout early afternoon and placed in 2mM 8-hydroxyquinoline (Soltis 1980) for up to 8 hours at 4°C or in N_2_O(Kato 1999) for 1 hour and then fixed in a 3:1 solution of absolute ethanol: glacial acetic acid for 2 to 24 hours. Root tips then were placed in 70% ethanol for at least 2 hours and digested in 40% HCl for 5-10 minutes (depending on the size of the root) at room temperature. Squashes were performed in 60% acetic acid and stained with 1% aceto-orcein dye and viewed on a Zeiss Photomicroscope III (Carl Zeiss, Oberkochen, Germany). To confirm each count, at least three to five metaphase cells were counted per specimen. These multiple counts per sample alleviated concerns regarding endomitosis, which has been reported in the allopolyploid (4*x*), *Opuntia spinosibacca* M.S. Anthony, 1956, (Weedin and Powell 1978), tetraploid *Opuntia pusilla* (Bowden 1945b), as well as in many other angiosperms (e.g., Barrow and Meister 2003, Tate et al. 2009, I. Jordan-Thaden, pers. comm.). We counted chromosomes of 277 individuals of the *Humifusa* clade, 14 individuals of *Opuntia stricta* s.l., three samples of the putative hybrid *Opuntia ochrocentra*, and two individuals of the putative hybrid *Opuntia alta* Griffiths, 1910. Generally, only one accession per population was counted.

*Taxonomy –* Taxa used for ploidy analysis are listed in Appendix 1. Species delimitations within *Opuntia humifusa* s.l. and *Opuntia macrorhiza* s.l. are problematic, so we recognize both *Opuntia humifusa* and *Opuntia macrorhiza* as broadly circumscribed (Table 1). Thus, we have arranged our counts of plants within these two species (see Appendix 1) according to their various segregates to determine whether the morphological variation of these segregate entities (Table 2) is correlated with cytotype and/or geographical and phylogenetic patterns.

**Table 1.** Synonyms of *Opuntia humifusa* s.l. and *Opuntia macrorhiza* s.l. sampled during this study.

*Opuntia humifusa* s.l.	*Opuntia macrorhiza* s.l.
*Opuntia allairei*	*Opuntia fusco-atra*
*Opuntia ammophila*	*Opuntia grandiflora*
*Opuntia austrina*	*Opuntia xanthoglochia*
*Opuntia cespitosa*	
*Opuntia lata*	
*Opuntia nemoralis*	
*Opuntia pollardii*	

**Table 2.** Selected taxa of *Opuntia humifusa* s.l. and *Opuntia macrorhiza* s.l. with morphological characters and corresponding ploidy. Polyploids often exhibit characters from more than one diploid taxon or characters of other polyploids, although certain characters (e.g., red glochids) have not been observed in any diploids analyzed thus far.

Taxon (ploidy)	Flower color	Cladode color	Spine barbedness/Cladode disarticulation	Glochid color
*Opuntia ammophila* (2x)	Yellow	Dark green	Not barbed/no	Stramineous
*Opuntia austrina* (2x)	Yellow	Dark green	Barbed/yes	Stramineous
*Opuntia cespitosa* (4x)	Red-centered	Glaucous green	Not barbed/no	Red
*Opuntia lata* (2x)	Yellow	Dark green	Barbed/yes	Stramineous
*Opuntia humifusa* (4x)	Yellow	Dark green	Not barbed/no	Stramineous
*Opuntia macrorhiza* (4x)	Red-centered	Glaucous green	Not barbed/no	Red/yellow
*Opuntia nemoralis* (4x)	Yellow	Glaucous green	Barbed/yes	Yellow
*Opuntia pollardii* (4x)	Yellow	Dark green	Barbed/yes	Stramineous
*Opuntia xanthoglochia* (2x)	Red-Centered	Glaucous green	Not barbed/no	Yellow

*Cytogeographic analysis –* We mapped the localities for all of the individuals for which we determined ploidy (277 in number) and incorporated previous counts (n = 41) (Bowden 1945a, Pinkava and McLeod 1971, Pinkava et al. 1973, Weedin and Powell 1978, Pinkava and Parfitt 1982, Pinkava et al. 1985, Weedin et al. 1989, Doyle 1990, Pinkava et al. 1992, Pinkava et al. 1998, Powell and Weedin 2001, Baker et al. 2009a, b; Majure and Ribbens in press) to cover the majority of the geographic distribution of each taxon. This allowed us to explore the geographic boundaries of the different ploidal levels encountered in this clade and construct hypotheses regarding polyploid formation and speciation.

*Phylogenetic analysis – *We generated sequences from the nuclear ribosomal internal transcribed spacer (nrITS: White et al. 1990) for a sample of diploid (n = 6) and polyploid taxa (n = 8) of the *Humifusa* clade from the eastern and western U.S. (Table 3). *Opuntia basilaris* Engelm. & J.M. Bigelow, 1856, was used as an outgroup based on previous analyses of *Opuntia* (LCM unpubl. data). A phylogenetic analysis of these data was carried out to determine whether the geographic distribution of ploidy (as determined here) was correlated with the evolutionary history of the clade. We carried out a Maximum Likelihood analysis using RAxML (Stamatakis 2006) running 10000 bootstrap pseudoreplicates under 25 rate categories and the GTR+Γmodel of molecular evolution.

**Table 3.** Taxa used in phylogenetic analyses of ITS sequence data given with their GenBank accession numbers.

Accession	Locality	GenBank accession #
*Opuntia basilaris* (outgroup)	Inyo Co., CA R. Altig s.n.	JF786913
*Opuntia abjecta* (2x)	Monroe Co., FL LCM 3908	JF787021
*Opuntia abjecta* (4x)	Monroe Co., FL LCM 3318	JQ245716
*Opuntia ammophila* (2x)	Marion Co., FL LCM 2826	JF786904
*Opuntia austrina* (2x)	Highlands Co., FL LCM 3450	JF786911
*Opuntia cespitosa* (4x)	Scott Co., MO LCM 2441	JQ245717
*Opuntia humifusa* (4x)	Warren Co., VA LCM 3800	JQ245718
*Opuntia lata* (2x)	Irvin Co., GA LCM 3785	JF786949
*Opuntia macrorhiza* (4x)	Kerr Co., TX LCM 3510	JF786960
*Opuntia nemoralis* (4x)	Garland Co., AR LCM 2196	JQ245720
*Opuntia pusilla* (2x)	Lowndes Co., MS LCM 843	JQ245721
*Opuntia pusilla* (3x)	Baldwin Co., AL LCM 1091	JF786985
*Opuntia pusilla* (4x)	Jackson Co., MS LCM 1920	JF786986
*Opuntia tortispina* (6x)	Hutchinson Co., TX LCM 3533	JF787020
*Opuntia xanthoglochia* (2x)	Bastrop Co., TX LCM 1982	JQ245719

## Results

**Figure 2.**
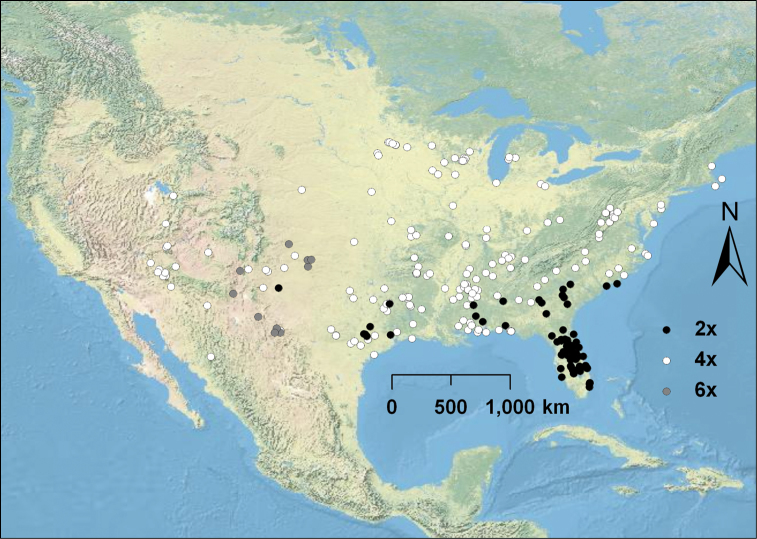
Cytogeography of *Opuntia humifusa* s.l., *Opuntia macrorhiza* s.l., *Opuntia pottsii*, and *Opuntia tortispina*. Diploids are represented with black circles, tetraploids by white circles, and hexaploids are represented by gray circles. *Opuntia humifusa* diploids are confined to the southeastern U.S., and *Opuntia macrorhiza* diploids are located in eastern Texas and southeastern New Mexico.

The base chromosome number for Cactaceae has been well established as *x* = 11 (Remski 1954, Pinkava and McLeod 1971, Lewis 1980, Pinkava et al. 1985, Pinkava 2002), and we saw no deviation from this in our counts (Appendix 1). Out of 318 counts of the *Humifusa* clade, including 41 from the literature, 210 (66%) were polyploid and 108 (34%) were diploid. Diploid (2*n* = 2*x* = 22) and tetraploid (2*n* = 4*x* = 44) *Opuntia humifusa* s.l. and *Opuntia macrorhiza* s.l. were discovered (Fig. 1A-D, I-J, L). Diploid *Opuntia humifusa* s.l. is restricted entirely to the southeastern U.S., whereas diploid *Opuntia macrorhiza* s.l. is restricted entirely to the southwestern U.S. (eastern Texas (see Appendix 1) and southeastern New Mexico (M. Baker and D.J. Pinkava pers. comm.)). Tetraploid members of *Opuntia humifusa* s.l. and *Opuntia macrorhiza* s.l. are much more widely distributed throughout the U.S. than are their diploid relatives (Fig. 2). Tetraploids of *Opuntia humifusa* s.l. are found from Massachusetts south to the southeastern U.S. where they abut the distribution of diploid taxa and throughout the eastern and midwestern U.S. Tetraploid *Opuntia macrorhiza* s.l. is distributed throughout parts of the Great Plains through the midwestern U.S., most of the southwestern U.S., parts of the Rocky Mountains, and the upper Sierra Madre Occidental in Sonora, Mexico (Fig. 2).

**Figure 3.**
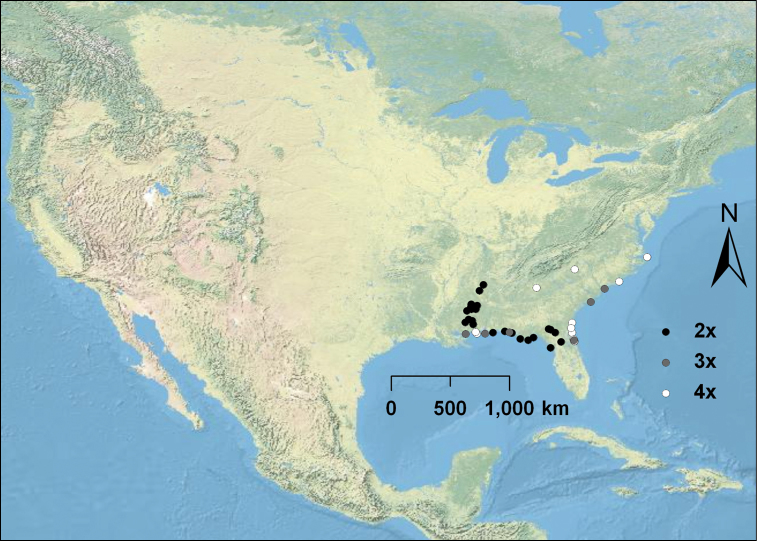
Cytogeography of *Opuntia pusilla*. Diploids are represented by black circles, triploids by gray circles, and tetraploids by white circles. Note that most polyploids are restricted to coastal areas.

Diploid, triploid, and tetraploid populations of *Opuntia pusilla* were discovered (Fig. 1E-G) throughout its restricted range in the southeastern U.S. (Fig. 3). Interestingly, with the exception of two populations, polyploid individuals (3*x* and 4*x*) were mostly confined to the coastline, although diploid populations were much more widespread throughout the interior part of the distribution of the species (Fig. 3). Of the three examples of *Opuntia abjecta* sampled from the Florida Keys, one was diploid (Fig. 1H), and two were tetraploid. *Opuntia tortispina* (southwestern U.S.) was hexaploid in six and tetraploid in one of the populations examined (see Fig. 2 for hexaploid distribution).

Individuals of *Opuntia stricta* sampled from the southeastern U.S. were all hexaploid. Samples included members of the taxa considered by some (Anderson 2001) to be *Opuntia dillenii* (Ker-Gawl.) Haw., 1819, and *Opuntia stricta*. Three individuals of the putative hybrid *Opuntia ochrocentra* from two localities in the Florida Keys were pentaploid (Fig. 1K), and the putative hybrid *Opuntia alta* was hexaploid.

**Figure 4.**
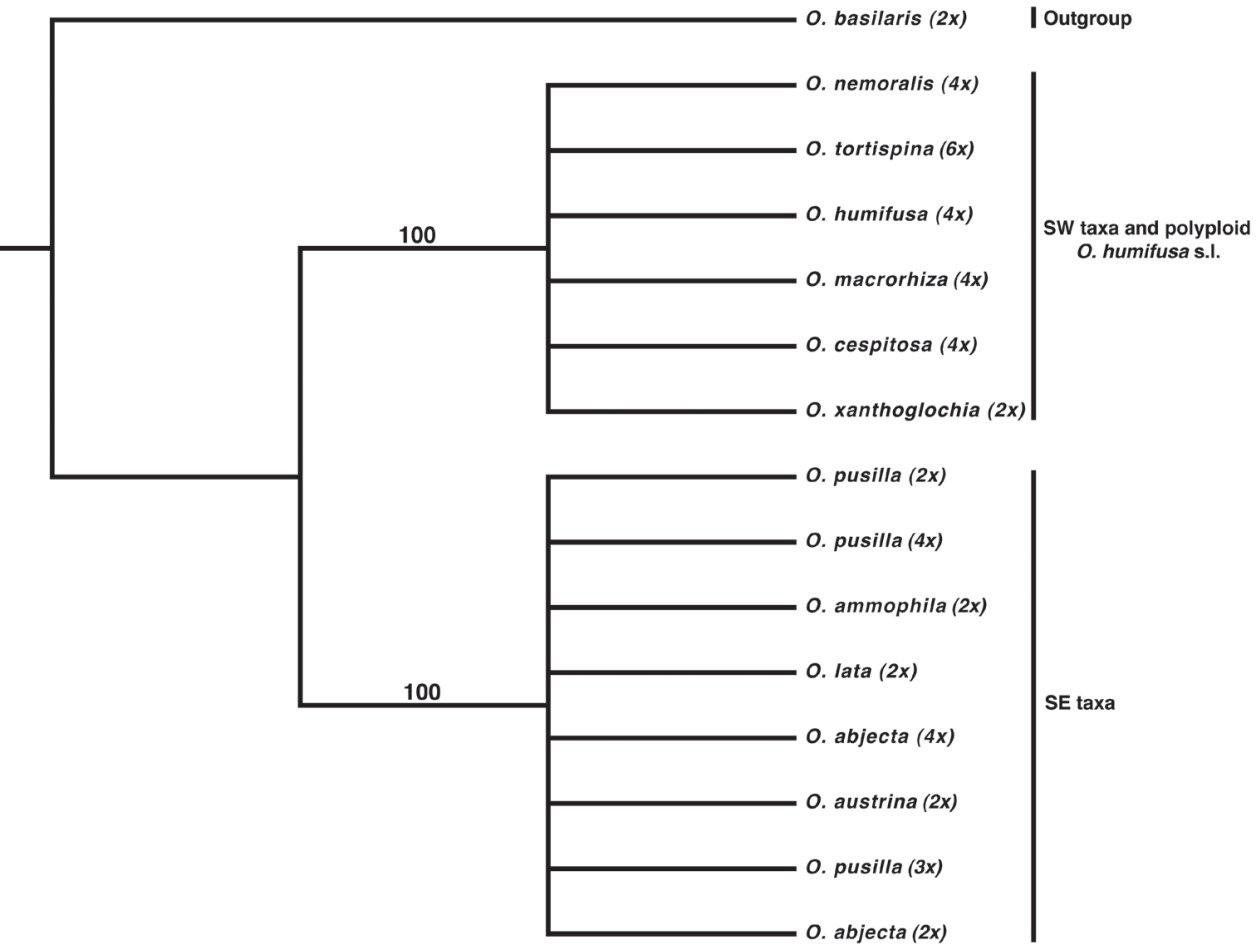
Majority rule consensus topology from 10000 ML bootstrap pseudoreplicates using RAxML based on the nrITS region. The western diploid *Opuntia macrorhiza* s.l. (*Opuntia xanthoglochia*) forms a well-supported clade with polyploid *Opuntia macrorhiza*, *Opuntia tortispina*, and the eastern polyploid morphotypes of *Opuntia humifusa* s.l. (*Opuntia cespitosa*, *Opuntia humifusa*, and *Opuntia nemoralis*). The southeastern diploid morphotypes of *Opuntia humifusa* s.l. (*Opuntia ammophila*, *Opuntia austrina*, *Opuntia lata*) and diploid *Opuntia abjecta* and *Opuntia pusilla* form a well-supported clade with polyploid members of *Opuntia pusilla* and *Opuntia abjecta*.

Maximum likelihood analysis of ITS data reveals that the *Humifusa* clade is made up of two well-supported subclades. One is restricted to the southeastern U.S. and includes polyploid members of *Opuntia pusilla* and *Opuntia abjecta*, and the other includes southwestern diploid *Opuntia macrorhiza* and all other polyploids pertaining to *Opuntia humifusa* s.l., *Opuntia macrorhiza* s.l., and *Opuntia tortispina*. There is no further resolution within the tree at the species level using ITS (Fig. 4). Species relationships within these two clades are further resolved with the addition of other loci (LCM unpubl. data), however, that is beyond the scope of this study.

## Discussion

*Opuntia macrorhiza* has only been recorded previously as tetraploid (Pinkava et al. 1971, 1973, 1977, 1992, 1998; Powell and Weedin 2001, 2004; Pinkava 2003). These are the first reports of diploid *Opuntia macrorhiza* and likely represent descendants of those progenitors from which tetraploid *Opuntia macrorhiza* s.l. and other polyploids arose. Likewise, this is the first report of diploid and triploid *Opuntia pusilla*, which was formerly known only from tetraploid counts (Bowden 1945a).

Diploid members of *Opuntia humifusa* s.l. (e.g., represented by the segregate taxa *Opuntia ammophila* Small, 1919, *Opuntia austrina* Small, 1903, *Opuntia lata* Small, 1919, in this study; see also Appendix 1) exhibit high levels of morphological variability but each is diagnosable morphologically, which suggests that these segregate taxa may need to be recognized at the species level. Likewise, diploid material of *Opuntia macrorhiza* s.l. from eastern Texas (e.g., *Opuntia xanthoglochia* Griffiths, 1910, in this study; see also Appendix 1) and southeastern New Mexico is morphologically distinct from tetraploid material of *Opuntia macrorhiza* s.l., which may also justify the recognition of *Opuntia xanthoglochia* and *Opuntia macrorhiza* as separate species.

Our hexaploid counts of *Opuntia stricta* are consistent with those of Pinkava et al. (1992) and Negrón-Ortiz (2007).In contrast,Spencer (1955) reported *Opuntia stricta* from Puerto Rico to be diploid. Other authors have also found Spencer’s counts from Puerto Rico to be inconsistent with more recent counts (e.g., Negrón-Ortiz 2007 for *Consolea* Lem., 1862).

Our three pentaploid counts of *Opuntia ochrocentra* support the proposed hybrid origin of this species between hexaploid *Opuntia stricta* (2*n* = 66) and diploid *Opuntia abjecta* (2*n* = 22) through unreduced gametes of *Opuntia abjecta*. *Opuntia ochrocentra* also exhibits intermediate morphological characters (e.g., growth form, spine characters) that further support its hybrid origin (LCM unpubl. data).

*Diploid refugia and polyploid formation* – Polyploidy is very common within the *Humifusa* clade, occurring in 66% of the samples reported here. Most researchers that have studied *Opuntia* cytologically have found polyploid taxa (e.g., Bowden 1945a, Weedin and Powell 1978, Pinkava et al. 1985, Doyle 1990, Segura et al. 2007, Baker et al. 2009a, b, Majure and Ribbens in press, but see Spencer 1955). All diploids in our analysis were restricted to either the southeastern or southwestern (eastern Texas and southeastern New Mexico) U.S., and the polyploid individuals were found nearly everywhere in between as well as north of these two diploid “refugia.” The disjunct pattern observed here in the *Humifusa* clade and in other studies between the southeastern U.S. and the southwestern U.S. is thought to have occurred as a result of the disruption of a semi-arid zone along the Gulf Coast region during the mid-Pleistocene (Webb 1990, Althoff and Pellmyr 2002). These two areas likely served as glacial refugia for a variety of animals and plants (e.g., Remington 1968, Davis and Shaw 2001, Al-Rabab’ah and Williams 2002, Althoff and Pellmyr 2002, Soltis et al. 2006, Waltari et al. 2007, Whittemore and Olsen 2011) and may have promoted current species richness and genetic diversity in southern populations (Hewitt 2000). Specifically, Swenson and Howard (2005) identified southeastern Texas and northern Florida as Pleistocene refugia for animal and plant species. Species from these regions subsequently came into contact following the last glacial maximum and formed hybrid zones at contact areas expanding out from these refugia. Swenson and Howard (2005) also hypothesized “post-glacial routes of expansion” from these proposed diploid refugia (e.g., Fig. 1, G & H in Swenson and Howard 2005). Those post-glacial routes and diploid contact zones are consistent with the current distributions of polyploid taxa within *Opuntia humifusa* s.l. and *Opuntia macrorhiza* s.l. The restricted diploid and widespread polyploid distribution pattern has been recorded in many other plants and is a common pattern seen in polyploid complexes (Babcock and Stebbins 1938, Stebbins 1950, 1971, DeWet 1971, Lewis 1980, Grant 1981, Parfitt 1991).

The seemingly disjunct southeastern New Mexico diploid population of *Opuntia macrorhiza* s.l. may represent a mere extension of the eastern Texas diploid refugium, which has since been mostly replaced by polyploid taxa. Alternatively, a diploid extension may still exist but was not detected due to the lack of cytological data for populations from east Texas to southeastern New Mexico (Fig. 2). Diploid taxa of other clades (e.g., *Opuntia polyacantha* Haw. var. *arenaria* (Engelm.) Parfitt, 1819) are coincidentally found near the same region (Pinkava 2002, 2003), however, suggesting that a third diploid refugium, i.e., in southeastern New Mexico-western Texas, may need to be recognized.

**Figure 5.**
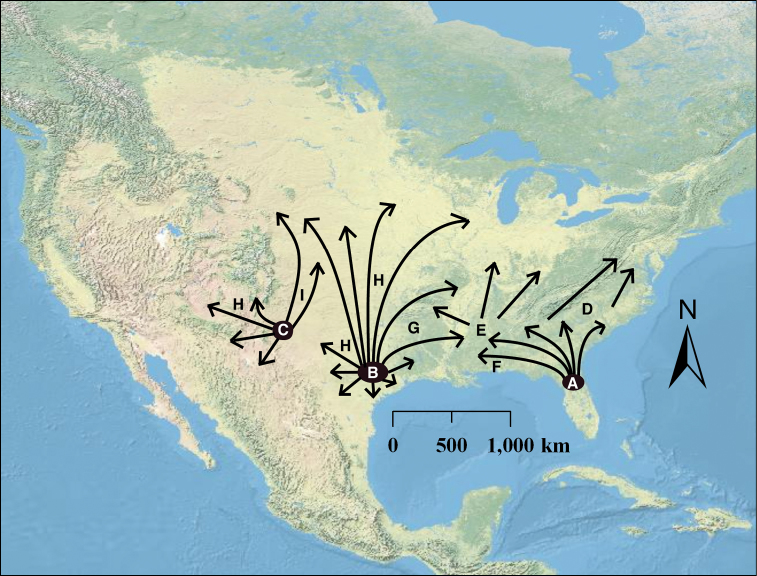
Hypothetical origin and subsequent dispersal of polyploid taxa from diploid refugia. Diploid refugia are represented by **A** southeastern *Opuntia humifusa* s.l. diploids **B–C** eastern Texas and southeastern New Mexico *Opuntia macrorhiza* s.l. diploids **D–I** represent polyploid formation where **D** represents *Opuntia humifusa* **E** represents *Opuntia cespitosa* **F** represents *Opuntia pollardii* **G** represents *Opuntia nemoralis* **H** represents tetraploid *Opuntia macrorhiza* (showing likely multiple formations), and **I** represents tetra- and hexaploid *Opuntia tortispina*.

Pinkava (2003) suggested that an *Opuntia humifusa* - *Opuntia macrorhiza* - *Opuntia pottsii* complex originated along the east coast of the U.S. and spread westward to Arizona, where it came into contact and hybridized with *Opuntia polyacantha* and formed the mostly hexaploid *Opuntia tortispina*. From our data, this scenario is plausible in that *Opuntia tortispina* has morphological characters representative of both *Opuntia polyacantha* and *Opuntia macrorhiza* and is found where populations of diploid and tetraploid *Opuntia macrorhiza* s.l. and diploid *Opuntia polyacantha* come into contact. However, considering the two diploid refugia suggested by our analyses and what is known about the historical biogeography of the southeastern U.S. (e.g., Webb 1990), it is likely that the *Humifusa* clade originated in the southwestern U.S. and adjacent northern Mexico, then dispersed eastward into the southeastern U.S. The arid habitat along the coast of the Gulf of Mexico during the mid-Pliocene to early Pleistocene would have been interrupted during the mid-Pleistocene, creating the disjunction and promoting the genetic divergence among diploid populations we see today (Fig. 4). Taxa from these two diploid refugia would have come back into contact and formed the widely successful polyploids of the Midwest and eastern U.S. (Fig. 5). This scenario is further corroborated by phylogenetic analyses, where eastern U.S. polyploids of *Opuntia humifusa* s.l. are resolved in a clade with the southwestern diploid *Opuntia macrorhiza* (Fig. 4). The lower frequency of diploids encountered in western populations of the *Humifusa* clade also suggest that those diploid populations may be older (see Stebbins 1971, p. 157) than those of the southeastern U.S.; however, this could merely be a bias resulting from more limited sampling of western populations.

The various morphotypes of tetraploid *Opuntia macrorhiza* in the western U.S. likely arose from southwestern diploid populations but subsequently spread in all directions after formation. Tetraploid *Opuntia macrorhiza* appears to have arisen numerous times, given that several morphotypes exist throughout its range. However, only two diploid morphotypes are known to exist (eastern Texas and southeastern New Mexico), suggesting that other ancestral diploids may have since gone extinct or have not yet been found, or that polyploid taxa exhibiting unique, derived characters were partly responsible for the origin of certain morphotypes, which have no diploid counterparts.

Stebbins (1971) suggested that there are several degrees of maturation of polyploid complex formation (i.e., initial, young, mature, declining, relictual), which may be deduced by comparing the relative geographic distribution of polyploids versus diploids. By these criteria, *Opuntia humifusa* s.l. and *Opuntia macrorhiza* s.l. may represent a mature polyploid complex. The diploid taxa are less common than polyploids and are largely restricted in distribution, whereas the polyploid taxa are much more widespread. Stebbins (1971) also proposed that mature polyploid complexes are relatively young, derived during the Plio- or Pleistocene epochs. This scenario would place polyploid formation in the *Humifusa* clade at the same time as Pleistocene megafauna. Thus, frequent environmental disturbances associated with glacial and interglacial cycles could have mediated the repeated contact of divergent diploid taxa leading to polyploid formation. Migrating herbivores would have then dispersed those polyploid products over large geographic areas (Jansen 1986). Divergence time estimation of the *Humifusa* clade places the origin of the clade in the late Pliocene to early Pleistocene (LCM, RP, PG, WSJ, PSS, DES unpubl. data), in agreement with this scenario. The occurrence of only polyploid individuals in previously glaciated areas of the U.S. provides further evidence for their subsequent spread into those available niches following the last glacial maximum.

Many polyploid populations of *Opuntia humifusa* s.l. and *Opuntia macrorhiza* s.l., especially in the eastern U.S., are largely isolated from one another and from diploid populations, suggesting that polyploid formation is not ongoing, at least on such a large scale as during the Pleistocene or immediately after the last glacial maximum. In contrast, polyploids in *Opuntia pusilla* are mostly sympatric with diploids in the Gulf of Mexico region and are represented by triploids and tetraploids. Polyploids of *Opuntia pusilla* also do not share the wide geographic distribution of those polyploids derived from *Opuntia humifusa* s.l. and *Opuntia macrorhiza* s.l. These observations suggest that the polyploids of *Opuntia pusilla* may have formed only recently, do not share comparable dispersal agents, or lack the obvious adaptive advantages of those polyploids derived from *Opuntia humifusa* s.l. and *Opuntia macrorhiza* s.l.

Many polyploid populations of *Opuntia humifusa* s.l. and *Opuntia macrorhiza* s.l.occupy northerly distributions and thus have a very high tolerance to cold temperatures. The hexaploid *Opuntia fragilis* (Nutt.) Haw., 1819 (not in the *Humifusa* clade) similarly inhabits areas of northern North America (Parfitt 1991, Loik and Nobel 1993, Ribbens 2008, Majure and Ribbens in press), with diploid relatives (e.g., *Opuntia polyacantha* var. *arenaria*) restricted to the southwestern U.S. (Parfitt 1991, Pinkava 2002). Thus, certain polyploid taxa appear to be more cold-resistant than their southerly diploid relatives (and presumed progenitors). *Opuntia humifusa* s.l. from northern areas of its distribution can withstand temperatures of -20°C (Nobel and Bobich 2002). However, the cold tolerance of diploid taxa has not been tested. Certain polyploid taxa of the *Humifusa* clade may therefore be better adapted to adverse environmental conditions than their diploid progenitors, which may partly explain their wide distribution relative to their diploid counterparts.

*Agamospermy* – The tetraploid *Opuntia cespitosa* (an entity within *Opuntia humifusa* s.l.; see Table 1) produces viable seed in the absence of outcrossing (Majure pers. obsv.), so this taxon is either self-compatible, which is common in Cactaceae (Rebman and Pinkava 2001), or agamospermous. Agamospermy is commonly associated with polyploidy (Stebbins 1950, DeWet and Stalker 1974, Harlan and DeWet 1975, Lewis 1980, Grant 1981, Whitton et al. 2008) and has been reported in numerous polyploid *Opuntia* species as well (Reyes-Agüero et al. 2006, Felker et al. 2010), including *Opuntia humifusa* s.l. and *Opuntia stricta* (Naumova 1993). Agamospermy would account for the high level of morphological variation observed among polyploid populations, as a result of the maintenance of a specific genotype within a given population through the lack of recombination (DeWet and Stalker 1974). Some agamic complexes also have wider distributions than their diploid progenitors (Babcock and Stebbins 1938, Stebbins 1950), as do certain polyploid taxa in this study.

*Autopolyploidy vs. Allopolyploidy* – The mechanism by which *Opuntia* polyploids are formed (auto- vs. allopolyploidy) is unclear. Unreduced gametes have frequently been found in meiotic analyses of Cactaceae (e.g., Pinkava et al. 1977, Pinkava and Parfitt 1982, Pinkava et al. 1985). Unreduced gamete formation coupled with interspecific hybridization (allopolyploidy) likely is a major factor in polyploid formation within the genus, given that *Opuntia* is renowned for hybridization (Benson 1982, Grant and Grant 1982, Pinkava 2002, Griffith 2004, LCM, RP, PG, WSJ, PSS, DES unpubl. data). It is probable that unreduced gamete formation within a single species (autopolyploidy) also plays a role in the formation of polyploids. Autopolyploids have been discovered in Cactaceae (Pinkava et al. 1985, Sahley 1996, Hamrick et al. 2002) and may be more common than is suspected.

*Opuntia humifusa* as currently circumscribed consists of numerous morphological entities, which are either diploid or tetraploid; those populations differing in ploidy are generally geographically well separated from one another. It is evident from our phylogenetic analysis (Fig. 4) that *Opuntia humifusa* is polyphyletic. Considering morphological and genetic data, it is likely that tetraploid *Opuntia humifusa* is of allopolyploid origin. However, the pattern in *Opuntia pusilla* is different, with populations of diploids found in close proximity to populations of triploids and tetraploids (Fig. 3). This evidence, plus morphological similarity among ploidal levels, suggests possible formation of autopolyploids. This same pattern is seen in other autopolyploid taxa (Lewis 1967, Nesom 1983), although there are exceptions to this pattern (Stebbins 1950, Soltis 1984, Husband and Schemske 1998). Molecular phylogenetic analysis (Fig. 4) and morphological characters (LCM, RP, PG, WSJ, PSS, DES unpubl. data; see Fig. 1E-G) of *Opuntia pusilla* also do not support an interspecific hybrid origin for the different ploidal levels herein observed for this species, although more variable molecular markers, cytogenetic work, and more detailed morphological analyses are needed to appropriately address this question.

*Morphological correlations with polyploids* – Some polyploid taxa in the *Humifusa* clade share morphological characters with diploids and other polyploids, suggesting that they may be derived from hybridization (Table 2). *Opuntia nemoralis* Griffiths, 1913, (Fig. 1J; an entity within *Opuntia humifusa* s.l.; see Table 1) shares spine color and orientation, cladode color, and glochid color of tetraploid *Opuntia macrorhiza* (from Arkansas), although, it possesses small and easily disarticulating cladodes, retrorsely-barbed spines, and the pile forming growth form and yellow flowers of *Opuntia pusilla* (Fig. 1E-G). *Opuntia cespitosa* (Table 1), as mentioned above, exhibits the red-centered flowers, glaucous-gray cladodes, and dark glochids (Fig. 1I) of tetraploid *Opuntia macrorhiza* (Fig. 1D), as well as the spine characters of diploid *Opuntia humifusa* s.l. (= *Opuntia ammophila*, *Opuntia austrina*, *Opuntia lata*; Table 2).

Throughout the distribution of the most common polyploid taxa, there also are polyploid populations that appear to be introgessive products of hybridization with other polyploids. For instance, in Michigan, Wisconsin, and western Illinois, certain populations display characters of both *Opuntia cespitosa* and tetraploid *Opuntia macrorhiza* (see Majure 2010, Fig. 1). In Bibb County, Alabama, populations appear to be intermediate between *Opuntia cespitosa* and *Opuntia pollardii* Britton & Rose, 1908,(tetraploids of *Opuntia humifusa* s.l.; see Table 1), with the red-centered flowers and rotund cladodes of *Opuntia cespitosa*, but the yellowish glochids and light green cladode color of *Opuntia pollardii.* In Fayette County, Tennessee, plants appear intermediate between *Opuntia humifusa* s.s. (i.e., tetraploid *Opuntia humifusa* represented by the type collection) and *Opuntia cespitosa*, having the yellowish glochids of tetraploid *Opuntia humifusa* s.s. and the spine characters of *Opuntia cespitosa*. Each one of the areas in which these intermediate plants occur appears to be a region of secondary contact, where polyploid taxa have introgressed to form new polyploid morphotypes that exhibit characters of both of the putative parents.

In the eastern U.S., most populations are represented by only one morphotype and thus appear to be morphologically stable (except for typically variable characters such as spine number; see Rebman and Pinkava 2001), indicating that hybridization is not ongoing among genomically distinct polyploid taxa. In contrast, in central Arkansas and populations farther west, more than one species and/or morphotype may be encountered within a given population. Also, in many coastal populations throughout the southeastern U.S., more than one species may be encountered, and putative hybrid taxa are sometimes observed.

## Conclusions

Members of the *Humifusa* clade are found throughout most of the continental U.S., with no obvious breaks or disjunctions in distribution patterns until detailed analyses of chromosome number were carried out. Our analyses indicate that diploid taxa in the *Humifusa* clade are presently confined to the southwestern and the southeastern U.S., which likely represent Pleistocene refugia for these taxa. Polyploid taxa of *Opuntia humifusa* s.l. and *Opuntia macrorhiza* s.l. were likely formed when diploids from these two refugia came into contact during interglacial cycles of the Pleistocene. This scenario is supported further by phylogenetic analyses, in which two clades correspond to these two diploid refugia, and polyploid taxa are found in either clade. Polyploid taxa likely also contributed to the diversity of polyploid morphotypes through secondary contact and introgression with other polyploids. After the end of the last glacial maximum, open niches would have been readily available for colonization by polyploid taxa produced towards the leading edge of the expansion and distribution of the *Humifusa* clade. These polyploids subsequently dispersed throughout most of the continent and occupied all suitable habitats available after glacial retreat, accounting for the distribution that we see today. Distributional success was enabled by the extreme cold tolerance displayed by many of the polyploid taxa, which allowed them to colonize more northern areas presumably unsuitable for diploid taxa.

## Appendix 1

Currently recognized *Opuntia* species investigated are listed (1-6). Synonyms of recognized species (sensu Benson 1982, Pinkava 2003, and Powell et al. 2008 in part; see Table 1) and their respective ploidy are given below the recognized species name. Recognized species are split by ploidy, where species have more than one cytotype. Their somatic chromosome number is given along with locality, collector, and repository according to Index Herbariorum (Thiers 2011). Taxa counted for the first time or cytotypes not previously recorded for a species are delimited with an asterisk (*). All counts were made by L.C. Majure.

**1) *Opuntia abjecta* Small**

*** *Opuntia abjecta* Small; 2*n* = 22 Florida,** Monroe Co., *LCM 3908* (FLAS). *** *Opuntia abjecta* Small; 2*n* = 44, Florida,** Monroe Co., *LCM 3318* (FLAS), Monroe Co., *KS s.n.* (FLAS).

**2) *Opuntia humifusa* (Raf.) Raf.**

***Opuntia humifusa* (2*x*) taxa: *Opuntia ammophila* Small; 2*n* = 22, Florida,** Brevard Co., *LCM 2087* (MISSA), Broward Co., *KS 62* (FLAS), Flagler Co., *LCM 3222* (FLAS), Indian River Co., *LCM 4182* (FLAS), Indian River Co., *LCM 4183* (FLAS), Indian River Co., *LCM 4184* (FLAS), Lake Co., *LCM 3246* (FLAS), Lake Co., *LCM 4093* (FLAS), Marion Co., *LCM 2753* (FLAS), Marion Co., *LCM 2754* (FLAS), Marion Co., *LCM 2826* (FLAS), Marion Co., *LCM 3247* (FLAS), Okeechobee Co., *LCM 4185* (FLAS), Okeechobee Co., *LCM 4186* (FLAS), Orange Co., *LCM 2086* (MISSA), Orange Co., *LCM 3962* (FLAS), Osceola Co., *LCM 3702* (FLAS), Osceola Co., *LCM 4181* (FLAS), Osceola Co., *LCM 4189* (FLAS), Putnam Co., *LCM 3248* (FLAS), St. Johns Co., *K.S. s.n.* (FLAS), St. Lucie Co., *LCM 3704* (FLAS), St. Lucie Co., *LCM 3705* (FLAS), St. Lucie Co., *LCM 3708* (FLAS), Seminole Co., *LCM 2085* (MISSA), Volusia Co., *LCM 3224* (FLAS), Volusia Co., *LCM 3232* (FLAS). ***Opuntia austrina* Small; 2*n* = 22, Florida,** Charlotte Co., *KS 45* (FLAS), Highlands Co., FL *KS 64* (FLAS), Highlands Co., *LCM 3450* (FLAS), Highlands Co., *LCM 3975* (FLAS), Highlands Co., *LCM 3976* (FLAS), Highlands Co., *LCM 3978* (FLAS), Okeechobee Co., *KS 29* (FLAS), Okeechobee Co., *KS 42* (FLAS), Palm Beach Co., *LCM 3970* (FLAS), Palm Beach Co., *LCM 3973* (FLAS), Polk Co., *KS s.n.* (FLAS), Polk Co., *LCM 3979* (FLAS). ***Opuntia lata* Small; 2*n* = 22, Alabama,** Autauga Co., *LCM 2043* (MISSA), Mobile Co., *LCM 4194* (FLAS), **Florida,** Alachua Co., *LCM 3991* (FLAS), Alachua Co., *LCM 4061* (FLAS), Alachua Co., *LCM 4064* (FLAS), Hernando Co., *LCM 3948* (FLAS), Highlands Co., *LCM 3977* (FLAS), Lafayette Co., *LCM 2795* (FLAS), Lake Co., *KS 15* (FLAS), Lake Co., *LCM 4117* (FLAS), Levy Co., *LCM 3645* (FLAS), Manatee Co., *LCM 4065* (FLAS), Okaloosa Co., *LCM 3954* (FLAS), Okeechobee Co., *LCM 4187* (FLAS), Okeechobee Co., *LCM 4188* (FLAS), Orange Co., *LCM 4174* (FLAS), Palm Beach Co., *LCM 3971* (FLAS), Putnam Co., *LCM 4106* (FLAS), Sumter Co., *LCM 3238* (FLAS), Sumter Co., *LCM 4066* (FLAS), **Georgia,**Charlton Co., *LCM 4190* (FLAS), Crawford Co., *JH s.n.* (FLAS), Irwin Co., *LCM 3785* (FLAS), Perry Co., *LCM 3786* (FLAS), Tatnall Co., *JH s.n.* (FLAS), **Mississippi,** Newton Co., *LCM 938* (MISSA), Wayne Co., *LCM 1290* (MISSA), **South Carolina,** Aiken Co., *LCM 3588* (FLAS), Horry Co., *LCM 3832* (FLAS).

***Opuntia humifusa* (4*x*) taxa: **Opuntia allairei* Griffiths; 2*n* = 44, Texas,** Liberty Co., *LCM 3504* (FLAS). ****Opuntia cespitosa* Raf.; 2*n* = 44, Alabama,** Bibb Co., *LCM 2042* (MISSA), Colbert Co., *LCM 2610* (MISSA),Lawrence Co., *LCM 2609* (MISSA), **Arkansas,**Garland Co., *LCM 2198* (FLAS), Garland Co., *LCM 4203* (FLAS), Garland Co., *LCM 4205* (FLAS), Saline Co., *LCM 2194* (MISSA), Yell Co., GPJ s.n. (FLAS), **Illinois,** Cass Co., IL *ER s.n.* (FLAS),Jo Daviess Co., IL *ER s.n.* (FLAS), **Kentucky,** Anderson Co., *LCM 3276* (FLAS), **Louisiana,** Caddo Parish, *LCM 4200* (FLAS), Caddo Parish, *LCM 4201* (FLAS), Caddo Parish, *LCM 4202* (FLAS), **Massachusetts,** Dukes Co., *BC s.n.* (FLAS), **Mississippi**, Lee Co., MS *JH s.n.* (FLAS), Lowndes Co., *LCM 755* (MISSA), Oktibbeha Co., *LCM 1380* (MISSA), Scott Co., *LCM 2563* (MISSA), **Tennessee,** Bledsoe Co., *LCM 1938* (MISSA), Cannon Co., *LCM 2072* (MISSA), Davidson Co., *JH s.n.* (FLAS), Fayette Co., *LCM 1956* (MISSA; note *Opuntia* cf. *cespitosa*), Fayette Co., *JH s.n.* (note *Opuntia* cf. *cespitosa* FLAS), Franklin Co., *BLS 2061* (FLAS), Lewis Co., *JH s.n.* (FLAS), Marshall Co., *JH s.n.* (FLAS), Rutherford Co., *JH s.n.* (FLAS), **Texas,** Lamar Co., *BS 2069* (FLAS), **Virginia,**Fredrick Co., *LCM 3806* (FLAS). ***Opuntia humifusa* (Raf.) Raf.; 2*n* = 44, Alabama,** Marion Co., AL *JH s.n.* (FLAS), **Delaware,** Sussex Co., *LCM 3824* (FLAS), **Georgia**,Dekalb Co., GA *LCM 3787* (FLAS), Jackson Co., *LCM 3789* (FLAS), Marion Co., *JH s.n.* (FLAS), **Maryland,** Alleghany Co., *LCM 3810* (FLAS), **Massachusetts**, Barnstable Co., MA *LCM 3814* (FLAS), **Mississippi,**Calhoun Co., MS *JH s.n.* (FLAS), Carroll Co, *LCM 799* (MISSA), Choctaw Co., *KP 499* (MMNS), Grenada Co., *LCM 1833* (MISSA), Marion Co., *JH s.n.* (FLAS), Marshall Co., *LCM 1293* (MISSA), Montgomery Co., *LCM 768* (MISSA), Stone Co., *TM s.n.* (FLAS), Webster Co., *KP 498* (MMNS), Yalobusha Co., *LCM 767* (MISSA), **New Hampshire,** Rockingham Co., *BN s.n.* (FLAS), **New Jersey,** Atlantic Co., *VD s.n.* (FLAS), Burlington Co., *LCM 3821* (FLAS), **North Carolina,** Bladen Co., *JH s.n.* (FLAS), Currituck Co., *LCM 3825* (FLAS), Dare Co., *LCM 3827* (FLAS), Onslow Co., *LCM 3829* (FLAS), Rowan Co., *LCM 3793* (FLAS), Surry Co., *JH s.n.* (FLAS), **South Carolina,** Pickens Co., *LCM 3790* (FLAS), York Co., *LCM 3791* (FLAS), **Virginia,**Fredrick Co., *LCM 3807* (FLAS), Page Co., *LCM 3799* (FLAS), Warren Co., *LCM 3800* (FLAS), **West Virginia,** Hampshire Co., *LCM 3808* (FLAS), Mineral Co., *LCM 3809* (FLAS), Pendleton Co., *ER s.n.* (FLAS). ****Opuntia nemoralis* Griffiths, 2*n* = 44, Arkansas,** Garland Co., *LCM 2192* (MISSA), Garland Co., *LCM 2196* (MISSA), Garland Co., *LCM 4204* (FLAS); **Louisiana,** Beauregard Parish, *CR s.n.* (FLAS), Cameron Parish, *LCM 4196* (FLAS), DeSoto Parish, *LCM 4198* (FLAS), Red River Parish, *LCM 4199* (FLAS), Winn Parish, *BLS 2053* (FLAS). ****Opuntia* cf. *nemoralis* Griffiths, 2*n* = 44, Arkansas,** Pulaski Co., *BLS 2131* (FLAS), Yell Co., TW s.n. (FLAS). ****Opuntia pollardii* Britton & Rose; 2*n* = 44, Alabama,** Baldwin Co., *LCM 1082* (MISSA), **Florida,**Santa Rosa Co., *LCM 1075* (MISSA), Walton Co., *LCM 1067* (MISSA), Walton Co., *LCM 1070* (MISSA), **Louisiana,** Washington Parish, *CR s.n.* (FLAS), **Mississippi,** Forrest Co., *LCM 806* (MISSA), Hancock Co., *LCM 748* (MISSA), Jackson Co., *LCM 1921* (MISSA), Jackson Co., *LCM 1297* (MISSA), Jackson Co., *LCM 4057* (FLAS), Jackson Co., *LCM s.n.* (MMNS), Neshoba Co., *LCM 1201* (MISSA), Noxubee Co., *LCM 1156* (MISSA), Stone Co., *TM s.n.* (FLAS), Winston Co., *LCM 769* (MISSA).

**3) *Opuntia macrorhiza* Engelm.**

***Opuntia macrorhiza* (2x) taxa: * *Opuntia xanthoglochia* Griffiths, 2*n* = 22, Texas,** Bastrop Co., *LCM 1982* (MISSA), Bastrop Co., *MJM 949* (FLAS), Fayette Co., *LCM 1983* (MISSA), Harris Co., *BLS 2089* (FLAS), Milam Co., TX *MJM 947* (FLAS),Smith Co., *BLS 2082* (FLAS).

***Opuntia macrorhiza* (4*x*) taxa: **Opuntia fusco-atra* Engelm.; 2*n* = 44, Texas,** Fayette Co., *LCM 3505* (FLAS). ****Opuntia grandiflora* Engelm.; 2*n* = 44, Arkansas,** Miller Co., *BLS 2062* (FLAS), **Mississippi,** Bolivar Co., *LCM 1680* (MISSA), Holmes Co., *HS s.n.* (FLAS), Yazoo Co., *LCM 2366* (MISSA), **Texas,** Anderson Co., *BLS 2077* (FLAS), Austin Co., *BLS 2091* (FLAS), Henderson Co., *BLS 2081* (FLAS), Jack Co., *LCM 3536* (FLAS), Leon Co., *BLS 2074* (FLAS), Marion Co., *BLS 2086* (FLAS), Smith Co., *LCM 3540* (FLAS), Van Zandt Co., *BLS 2083* (FLAS). ***Opuntia macrorhiza* Engelm., 2*n* = 44, Arkansas,** Nevada Co., *BLS 2130* (FLAS), Newton Co., *MC s.n.* (FLAS), Pulaski Co., *LCM 4206* (FLAS), **Arizona,** Coconino, *TH s.n.* (FLAS), Coconino, *BW s.n.* (FLAS), **Nebraska,** Keith Co.,NE *ER s.n.* (FLAS),Lancaster Co., *TH s.n.* (FLAS), **New Mexico,** Torrance Co., *LCM 3530* (FLAS), **Texas,**Calhoun Co., TX *MJM 962* (FLAS),Dallas Co., *LCM 3539* (FLAS),Gonzales Co., *MJM 958* (FLAS), Kimble Co., *LCM 3511* (FLAS), Kerr Co., *LCM 3508* (FLAS), Kerr Co., *LCM 3510* (FLAS),Palo Pinto Co., *LCM 3537* (FLAS), **Utah,** Salt Lake Co., *TH s.n.* (FLAS), Sevier Co., *TH s.n.* (FLAS).

**4) *Opuntia pusilla* (Haw.) Haw.**

*** *Opuntia pusilla*, 2*n* = 22, Alabama,** Lamar Co., *JH s.n.* (FLAS), **Florida,** Alachua Co., *LCM 4003* (FLAS), Bay Co., *KS 307* (FLAS), Bay Co., *KS 309* (FLAS), Columbia Co., *LCM 4191* (FLAS), Escambia Co., *KS 328* (FLAS), Franklin Co., *KS 301* (FLAS), Franklin Co., *KS 330* (FLAS), Gulf Co., *KS 325* (FLAS),Hamilton Co., *LCM 4192* (FLAS), Hamilton Co., FL *LCM 4193* (FLAS), Levy Co., *LCM 2819* (FLAS), **Mississippi,** Clarke Co., *LCM 1270* (MISSA), Forrest Co., *LCM 756* (MISSA), Jasper Co., *LCM 766* (MISSA), Lamar Co., *LCM 1548* (MISSA), Lauderdale Co., *LCM 2094* (MISSA), Lauderdale Co., *LCM 3919* (MISSA), Lowndes Co., *LCM 843* (MISSA), Newton Co., *LCM 828* (MISSA), Newton Co., *LCM 937* (MISSA), Newton Co., *LCM 4211* (FLAS), Perry Co., *LCM 757* (MISSA), Smith Co., *LCM 753* (MISSA), Wayne Co., *TM s.n.* (FLAS), Wayne Co., *TM s.n.* (FLAS). *** *Opuntia pusilla*, 2*n* = 33, Alabama,** Baldwin Co., *LCM 1091* (MISSA), **Florida,** Flagler Co., *LCM 3221* (FLAS), St. Johns Co., *LCM 3219* (FLAS), Walton Co., *LCM 1066* (MISSA), **Mississippi,** Hancock Co., *LCM 1033* (MISSA), **South Carolina,** Horry Co., *JH s.n.* (FLAS), Horry Co., *LCM 3833* (FLAS). ***Opuntia pusilla*, 2*n* = 44, Florida,** Duval Co., *LCM 3700* (FLAS), Nassau Co., *CJ s.n.* (FLAS), St. Johns Co., *LCM 3218* (FLAS), St. John’s Co., *KS 9.4.10* (FLAS), **Georgia,**Dekalb Co., *LCM 3788* (FLAS), Glynn Co., *TM s.n.* (FLAS), **Mississippi,** Jackson Co., *LCM 955* (MISSA), Jackson Co., *LCM 1920* (MISSA), **North Carolina,** Dare Co., *LCM 3828* (FLAS), Dare Co., *LCM 3836* (FLAS), New Hanover Co., *LCM 3830* (FLAS), **South Carolina,** York Co., *LCM 3792* (FLAS).

**5a) *Opuntia stricta* (Haw.) Haw.**

***Opuntia dillenii* (Ker-Gawl.) Haw., 2*n* = 66, Florida,** Charlotte Co., *LCM 3949* (FLAS), Flagler Co., *LCM 3220* (FLAS), Monroe Co., *LCM 3319* (FLAS), Hillsborough Co., *LCM 3952* (FLAS), **Puerto Rico,** Cabo Rojo, *LCM 3843* (FLAS). ***Opuntia stricta* (Haw.) Haw., 2*n* = 66, Alabam*a,***Mobile Co., *LCM 823* (MISSA), **Florida,** Clay Co., *LCM 3701* (FLAS), Levy Co., *LCM 2820* (FLAS), Monroe Co., *LCM 3320* (FLAS), St. Johns Co., *LCM 3217* (FLAS), Seminole Co., *LCM 2083* (MISSA), **Mississippi,** Jackson Co., *LCM 1922* (MISSA).

**5b) Putative hybrids involving *Opuntia stricta*.**

***Opuntia alta* Griffiths 2*n* = 66, Louisiana,** Cameron Parish, *LCM 4195* (FLAS), LaFourche Parish, *CR s.n.* (FLAS). *** *Opuntia ochrocentra* Small, 2*n* = 55, Florida,** Monroe Co., *LCM 3907* (FLAS), Monroe Co., *LCM 3968* (FLAS), Monroe Co., *LCM 3969* (FLAS).

**6) *Opuntia tortispina* Engelm. & J.M. Bigelow.**

***Opuntia tortispina*, 2*n* = 44, New Mexico,** Quay Co., *LCM 3531* (FLAS), ***Opuntia tortispina*, 2*n* = 66**, **New Mexico,** Benalillo Co., *LCM 3528* (FLAS), Sierra Co., *LCM 3521* (FLAS), **Oklahoma,** Cimarron Co., *ER s.n.* (FLAS), **Texas,** Carson Co., *LCM 3532* (FLAS), Hutchinson Co., *LCM 3533* (FLAS), Hutchinson Co., *LCM 3535* (FLAS).
